# Stillbirth rate trends across 25 European countries between 2010 and 2021: the contribution of maternal age and multiplicity

**DOI:** 10.1093/eurpub/ckae214

**Published:** 2025-01-21

**Authors:** Maxi S Kniffka, Jonas Schöley, Susie Lee, Loes C M Bertens, Jasper V Been, Jóhanna Gunnarsdóttir, The Euro-Peristat Network, Alex Farr, Jeannette Klimont, Sophie Alexander, Wei-Hong Zhang, Gisèle Vandervelpen, Rumyana Kolarova, Evelin Yordanova, Željka Draušnik, Theopisti Kyprianou, Vasos Scoutellas, Petr Velebil, Laust Hvas Mortensen, Luule Sakkeus, Liili Abuladze, Mika Gissler, Anna Heino, Béatrice Blondel, Catherine Deneux-Tharaux, Mélanie Durox, Alice Hocquette, Marianne Philibert, Jennifer Zeitlin, Jeanne Fresson, Guenther Heller, Bjoern Misselwitz, Aris Antsaklis, István Sziller, Johanna Gunnarsdottir, Helga Sól Ólafsdóttir, Karen Kearns, Izabela Sikora, Marina Cuttini, Marzia Loghi, Serena Donati, Rosalia Boldrini, Janis Misins, Irisa Zile, Jelena Isakova, Aline Lecomte, Audrey Billy, Jessica Pastore, Daniel Alvarez, Miriam Gatt, Jan Nijhuis, Lisa Broeders, Peter Achterberg, Ashna Hindori-Mohangoo, Kari Klungsoyr, Rupali Akerkar, Hilde Engjom, Katarzyna Szamotulska, Ewa Mierzejewska, Henrique Barros, Mihai Horga, Vlad Tica, Mihaela-Alexandra Budianu, Alexandra Cucu, Cristian Calomfirescu, Jan Cap, Miha Lučovnik, Ivan Verdenik, Oscar Zurriaga, Adela Recio Alcaide, María Fernández Elorriaga, Mireia Jané, Maria José Vidal, Karin Källén, Anastasia Nyman, Tonia Rihs, Alison Macfarlane, Sonya Scott, Kirsten Monteath, Lucy Smith, Ruth Matthews, Siobhán Morgan, Joanne Murphy

**Affiliations:** Max Planck Institute for Demographic Research, Social Demography, Rostock, Germany; Department of Obstetrics and Gynaecology, Erasmus MC Sophia Children’s Hospital, University Medical Centre Rotterdam, Rotterdam, Netherlands; Max Planck Institute for Demographic Research, Social Demography, Rostock, Germany; Max Planck Institute for Demographic Research, Social Demography, Rostock, Germany; Department of Obstetrics and Gynaecology, Erasmus MC Sophia Children’s Hospital, University Medical Centre Rotterdam, Rotterdam, Netherlands; Department of Obstetrics and Gynaecology, Erasmus MC Sophia Children’s Hospital, University Medical Centre Rotterdam, Rotterdam, Netherlands; Department of Neonatal and Paediatric Intensive Care, Erasmus MC Sophia Children’s Hospital, University Medical Centre Rotterdam, Rotterdam, Netherlands; University of Iceland, Faculty of Medicine, Reykjavik, Iceland; Landspitali—The National University Hospital of Iceland, Department of Obstetrics and Gynecology, Reykjavik, Iceland

## Abstract

Stillbirth rates have stalled or increased in some European countries during the last decade. We investigate to what extent time-trends and between-country differences in stillbirth rates are explained by the changing prevalence of advanced maternal age and teenage pregnancies or multiple births. We analysed data on stillbirths and live births by maternal age and multiplicity from 2010 to 2021 in 25 European countries using Kitagawa decomposition to separate rate differences into compositional and rate components. Rates significantly decreased in six countries, but increased in two. Changes in maternal age structure reduced national stillbirth rates by a maximum of 0.04 per 1000 in the Netherlands and increased rates by up to 0.85 in Cyprus. Changes in the prevalence of multiple births decreased rates by up to 0.19 in the Netherlands and increased rates by up to 0.01 across multiple countries. Maternal age differences explained between 0.11 of the below-European average stillbirth rate in Belgium and 0.13 of the above-average rate in Ireland. Excluding Cyprus, differences in multiple births explained between 0.05 of the below-average rate in Malta and 0.03 of the above-average rate in Ireland. For most countries, the increase in advanced-age pregnancies contributed to rising stillbirth rates over time, while reductions in multiples led to decreases in rates. However, large parts of the trends remain unexplained by those factors. By 2021, neither factor explained the differences between countries, due to increased compositional uniformity and declining stillbirth risk for advanced maternal age.

## Introduction

In Europe, there has been a historical trend of decreasing rates of stillbirth (foetal death in late pregnancy) over several decades. While the majority of European countries have continued to reduce stillbirth rates to very low levels of less than 3 per 1000 births [1], stillbirth rates have tended to plateau or even increase in several other countries [2–6], with the COVID-19 pandemic being discussed as a contributing factor since 2020 [7–9]. We aim to investigate how variations in higher-risk pregnancies, influenced by maternal age and multiple pregnancies, contribute to the different trends observed across European countries and the disparities between countries.

Advanced maternal age [10, 11] and multiple pregnancies [12] are two important demographic factors associated with stillbirth risk which are routinely recorded in birth registries. The risk of stillbirth is slightly increased in younger mothers but rises more steeply after the age of 34 years [10, 11]. Advanced maternal age may increase stillbirth risk due to a higher prevalence of maternal morbidity and obstetric complications, such as pregnancy-induced hypertensive disorders and placental or cervical insufficiency [13, 14], and pre-pregnancy conditions such as diabetes and hypertension [15]. Pregnancies at an advanced age may also modify the effect of toxic exposures such as smoking [16]. The risk of stillbirth is around 3 times higher in multiple pregnancies than in singleton pregnancies [17]. This can be explained in part by the risk of twin-to-twin transfusion syndrome or a higher prevalence of congenital malformations, pre-eclampsia, and placental insufficiency [18].

Importantly, the population-level prevalence of these risk factors has changed in recent decades. The average age of mothers at birth has increased in European countries due to delayed childbearing, a prominent demographic change over the last century [1]. Multiple birth rates have also increased dramatically in high-income countries, but rates have recently stagnated in some countries due to changes in multiple births conceived through IVF [19]. These changes imply that the population distribution of demographic risk factors for stillbirth also changed. For example, it is possible that the recent increase in stillbirth rates in some European countries is the compositional effect of more older mothers being pregnant rather than an increase in the individual risk of stillbirth itself.

The changing maternal age structure in the USA has been shown to account for one-third of the decline in national rates [20]. A study from Germany also described a relationship between increasing maternal age and changes in stillbirth rates [21], but did not analyse the extent to which national stillbirth rates are influenced by this compositional factor.

The proportion of multiple pregnancies varies considerably over time within countries in Europe, but even more so between countries [1]. These differences also have the potential to explain population-level differences in stillbirth rates. This is especially important, as modern techniques in assisted reproduction, like elective single embryo transfer, can reduce the rate of multiple pregnancies, but are used to different extents across countries.

The present study extends the existing literature by first providing a comparative overview of different patterns of stillbirth trends across European countries, accompanied by an overview of how maternal age structure and multiplicity have changed over time. Finally, we use a demographic decomposition technique to analyse how changes in maternal age and multiplicity contribute to the differences in stillbirth rates. We look at these relationships over time, from 2010 to 2021. Additionally, we examine how national stillbirth rates in 2021 differ from the European average.

## Methods

### Data

**Figure 1.**
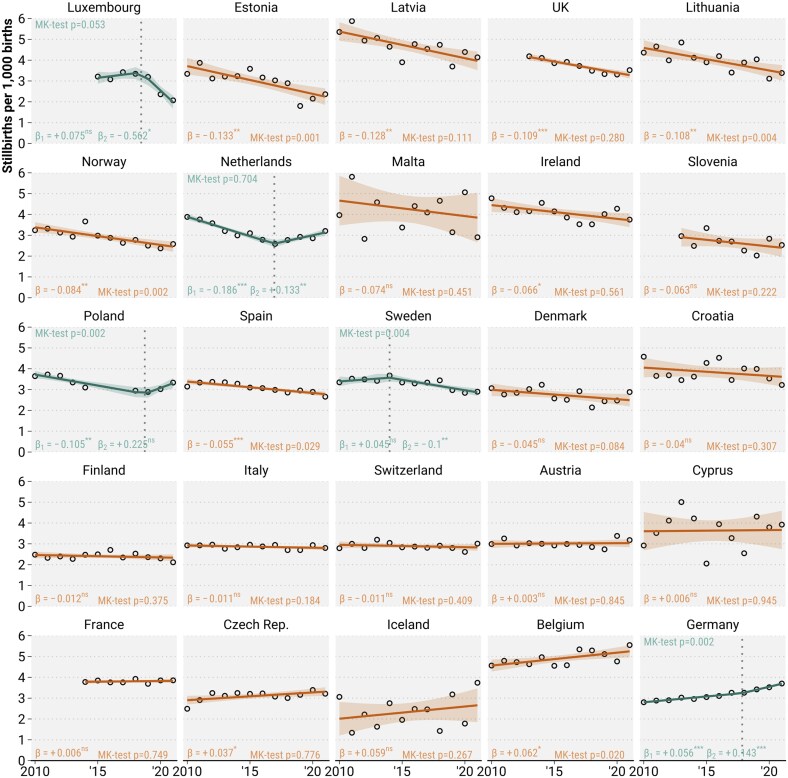
Trends in stillbirth rates between 2010 and 2021 across 25 European countries. The dots mark the annual rates (2013–2021 for the UK and Slovenia, 2014–2021 for France, and 2015–2021 for Luxembourg). Slope estimates of the trend in stillbirth rates originate either from a simple linear regression (orange) or, if a significant breakpoint was identified, from a segmented regression (green). Mean predictions and 95% confidence intervals from the regression fit are superimposed. MK test for monotonic trend with correction for autocorrelation. *P < .05, **P < .01, ***P < .001. Source: Euro-Peristat Network.

A stillbirth is defined as the birth of a baby with no signs of life. Most stillborn babies die before labour begins, but some of these deaths occur during labour [22]. We used data on the annual aggregated number of stillbirths and live births from 2010 to 2021 for 25 out of the 31 countries from the Euro-Peristat project [1], who were able to provide the needed data (Fig. 1). Data from the Euro-Peristat Network comes from routine sources, including vital statistics, birth registers, hospital discharge data, and routine surveys [1]. To avoid issues with small numbers, the UK was analysed as a whole. Detailed information on stillbirths by maternal age and multiplicity was available for 2010, 2015, and 2021 (for Poland 2010, 2014, and 2021). For Luxembourg, data was available from 2015, for France from 2014, and for the UK and Slovenia from 2013. For Spain, stillbirths by multiplicity were available from 2015. Luxembourg was excluded from the decomposition analysis because only annual data was available, without information on maternal age and multiplicity. Due to data protection guidelines, stillbirths by maternal age or multiplicity were not available fully cross-tabulated. Thus, the decomposition analyses were done separately for the two strata.

To obtain comparable results, a consistent definition of stillbirth is crucial. The Euro-Peristat project provides an excellent database for this analysis, using a nearly uniform definition of stillbirth at 24 weeks’ gestation. Since most countries register stillbirths from either 22 or 24 weeks’ gestation or a birth weight of at least 500 g, 24 weeks is the earliest threshold to include data from all countries. Wherever possible, termination of pregnancy (TOP) has been excluded from the data. In two cases, cell counts with very low numbers were not given exactly and were randomly imputed. Some countries had missing information on maternal age and multiplicity, which was redistributed proportionally within existing groups for stillbirths and live births, assuming randomness in the missing data. This redistribution, rather than exclusion, prevented bias in our results by ensuring that no stillbirths were excluded in regions with lower data completeness.

### Analysis

Rates were calculated as stillbirths per 1000 births [23]. The Mann–Kendall (MK) test with correction for autocorrelation was used to test for monotonic trends in the time series of national rates [24]. The average annual change in stillbirth rates was estimated via (segmented) linear regression. If a significant breakpoint was identified in the time series (*P* < .05, score test for change in slopes) [25], we reported slopes pre and post the breakpoint; otherwise, we reported a single slope. To identify possible effects of the COVID-19 pandemic, we employed robust standardized residuals to test if the stillbirth rates in the years 2020 and 2021 were unusually high [26].

We used the Kitagawa decomposition [27] to separate the stillbirth rate differences into compositional and rate components:


$$\Delta =\sum_{i}^{}\left(C_{i}^{B}-C_{i}^{A}\right)\times \left[\frac{M_{i}^{B}+M_{i}^{A}}{2}\right]+\sum_{i}^{}\left(M_{i}^{B}-M_{i}^{A}\right)\times \left[\frac{C_{i}^{B}+C_{i}^{A}}{2}\right]$$

where *A* and *B* index the two populations which yield the rate difference Δ (time points A and B, country A and cross-country average B), C_i_ is the share of the total births in stratum *i* (maternal age, multiplicity), and M_*i*_ is the stillbirth rate in the respective stratum.

The Kitagawa decomposition is based on counterfactual prediction of rates. The compositional difference is the difference in rates if only the proportions of population strata would differ among the two populations but the within-stratum rates were equal. Conversely, the rate difference is contributed by differences in rates within the population strata of two populations, assuming equal stratum composition. Both differences add to the total rate difference.

We decomposed the change in stillbirth rates between 2010 and 2021 and calculated the extent to which the change in population composition, either by maternal age or multiplicity, was responsible for the change in stillbirth rates. This result is called the composition effect. The complementary rate effect tells us how the total stillbirth rate would have changed if the composition of the population along multiplicity and maternal age had remained the same.

For 2021, we decomposed the difference between national stillbirth rates and the European average, which we calculated by summing up stillbirths and births across all 25 countries.

## Results

### Changes in stillbirth rates, maternal age structure, and multiple pregnancy rates

Figure 1 shows the national trends in stillbirth rates from 2010 to 2021. Levels differ between countries, ranging from 2.1 in Finland to 5.5 per 1000 in Belgium in 2021. We identified a linear decrease in stillbirth rates in 14 out of 25 countries and a non-linear decrease in four countries. The declining trends were significant (Supplementary Table S1, *P*-values of MK trend test <.05) for Estonia, Lithuania, Norway, Poland, Spain, and Sweden. Rates were increasing in seven countries. Germany and Belgium show a significantly rising trend in stillbirth rates, with an annual increase of 0.056 until 2018 and 0.143 thereafter for Germany and 0.062 per 1000 for Belgium. With the exception of Belgium, all countries with a stillbirth rate above 4.0 per 1000 in 2010 experienced an annual decrease in stillbirth rates (0.066–0.128 per 1000).

We found no evidence that European stillbirth rates were systematically elevated over 2020 and 2021. Stillbirth rates in 2020 were below their expectation in 12 out of 25 countries, and likewise in 8 out of 25 countries in 2021. Unusually elevated stillbirth rates since 2020 (*z*-score > 2) were found for the UK (*z* = 2.685), the Netherlands in 2021 (*z* = 2.083), and Austria in 2020 (*z* = 2.766). See Supplementary Fig. S1 for complete depiction of the robust standardized residuals.

**Figure 2.**
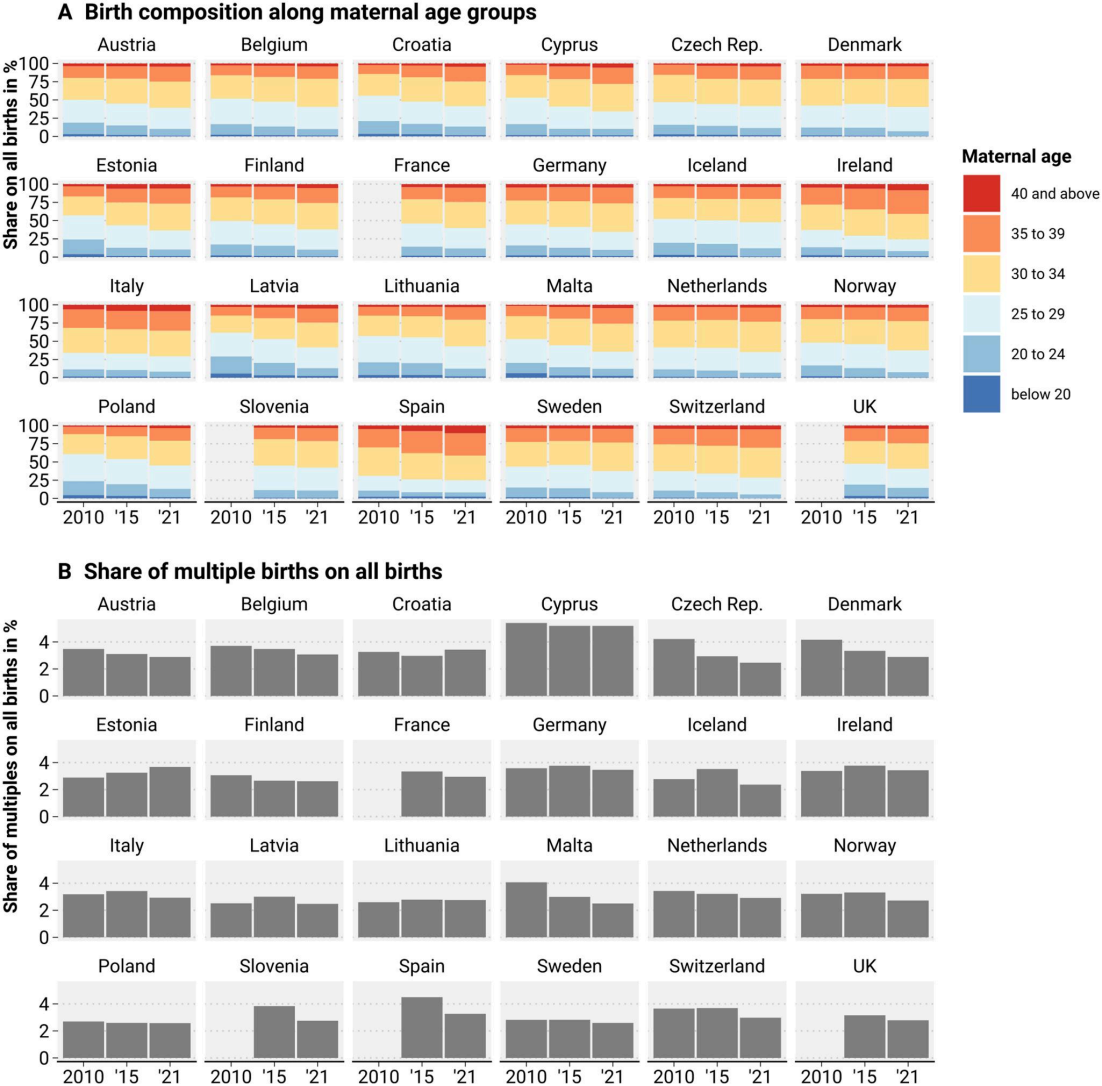
Change in the composition of births across 24 European countries between 2010 and 2021. (A) Maternal age structure from 2010 to 2021. (B) Births from multiple pregnancies per 100 total births from 2010 to 2021. Source: Euro-Peristat Network.

Since 2010, the prevalence of advanced maternal age at birth and multiple pregnancies has changed. The average age of mothers has increased in all countries observed, with fewer mothers aged under 25 and more mothers aged 35 and over (Fig. 2A). Ireland, Italy, and Spain have the highest proportions of mothers aged 35 or over, while the proportion of young mothers aged under 25 is higher in Estonia, Latvia, Lithuania, Malta, and Poland, especially in 2010. The prevalence of multiple births ranges from 2.4% in Iceland to 5.4% in Cyprus (Fig. 2B). Most countries show a decrease in multiple births over time. Between 2010 and 2021, the stillbirth risk improved the most for maternal ages over 35 and 40 but increased slightly for the youngest age group (Supplementary Fig. S2).

### Decomposition of stillbirth rates over time

**Figure 3.**
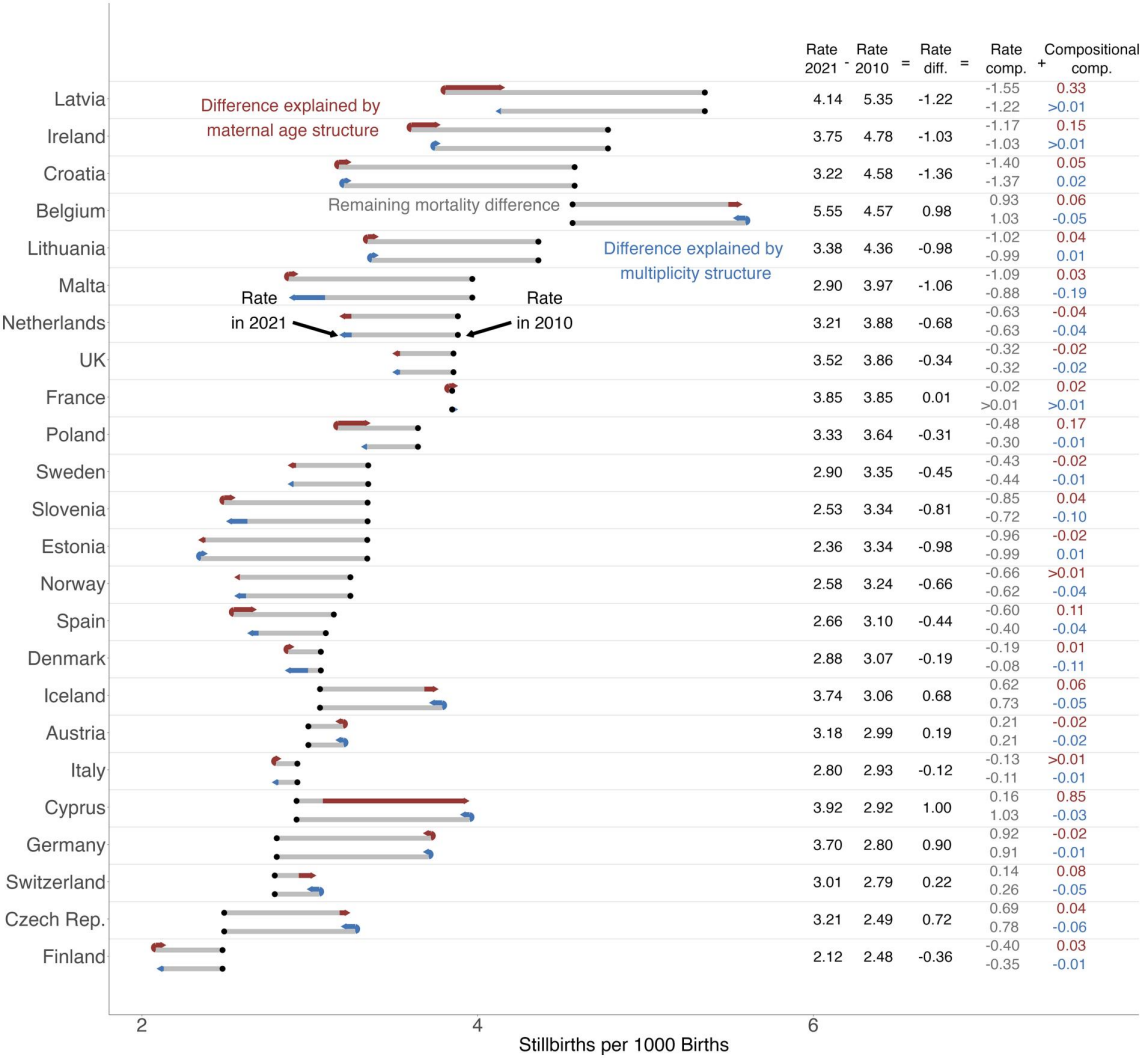
Decomposition of the difference in stillbirth rates by country between 2010 and 2021 into the component of changing stillbirth rates (grey), changing maternal age composition (red), and changing prevalence of multiples (blue). Decomposition of maternal age structure and multiples were performed separately. The black dot represents the rate in 2010 [2015 for the UK, France, and Slovenia (age and multiples) and Spain (multiples)] and the end of the arrow represents the rate in 2021. Source: Euro-Peristat Network.

Figure 3 shows the result of decomposing stillbirth rates over time between 2010 (black dot) and 2021 (arrow) for each country separately. The effect of the changing age structure is shown in red and is positive for most countries, meaning that the increase in maternal age alone would have resulted in an increase in the national stillbirth rate if age-specific stillbirth rates had remained constant. In Cyprus, for example, the stillbirth rate increased by about 1 from 2.9 to 3.9 per 1000. However, the age structure alone would have increased the rate by 0.85, while the remaining increase of 0.16 per 1000 was due to unobserved factors. This suggests that most of the increase was due to changes in the age structure alone.

**Figure 4.**
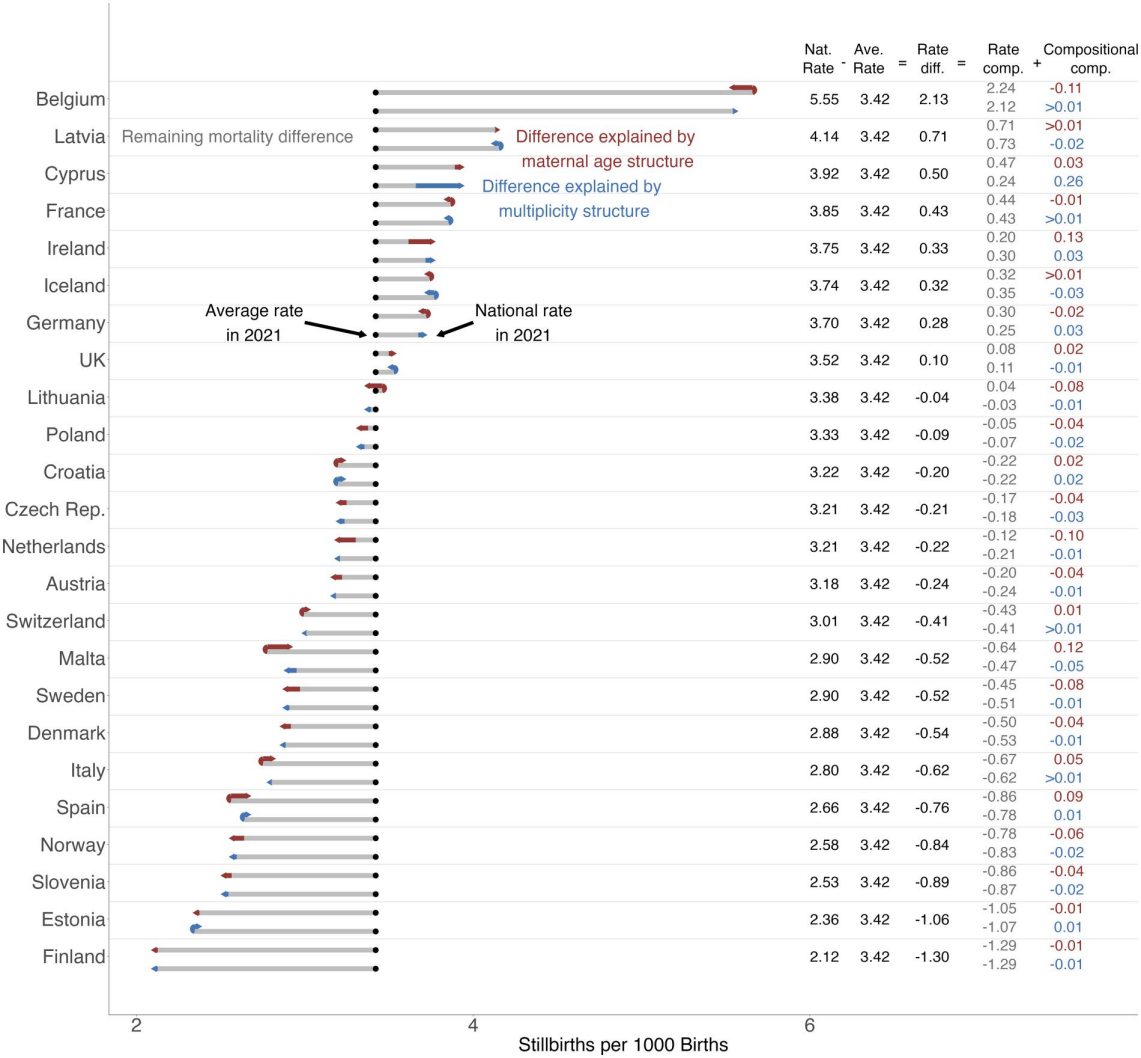
Decomposition of the difference in stillbirth rates between countries and European average in 2021 into the component of changing stillbirth rates (grey), changing maternal age composition (red), and changing prevalence of multiples (blue). Decomposition of maternal age structure and multiples were performed separately. The black dot represents the average rate in 2021 across all countries, and the end of the arrow represents the national rate in 2021. Source: Euro-Peristat Network.

In countries with an overall decrease in stillbirth rates, such as Latvia (−1.22 per 1000), the increase in maternal age would also have led to a higher national stillbirth rate, increasing the rate by 0.33 per 1000. However, age-specific rates had the opposite effect, as they fell between 2010 and 2021, resulting in a decrease in the national rate by 1.55 per 1000, assuming a constant age structure. Thus, if the age structure had not changed, the national stillbirth rate in Latvia would have been even lower than observed in 2021. Overall, the effect size of the change in maternal age structure ranged from a decrease of 0.04 in the Netherlands to an increase of 0.85 per 1000 in Cyprus.

The effect of the changing proportion of multiple births is shown in the blue bars. For most countries, the effect of the change in the proportion of multiple births was negative, meaning that the national stillbirth rate would have decreased solely due to a reduction in multiple births. In Denmark, for example, we observed a small decrease in the national stillbirth rate of 0.19 per 1000. The change in multiplicity alone would have reduced the stillbirth rate by 0.11 per 1000, which means that most of the reduction was due to the decrease in multiple births over time. The effect size of the change in multiplicity structure ranged from a decrease in national rates of 0.19 in Malta to an increase of 0.01 per 1000 in several countries. These small effect sizes imply that, overall, the changing proportion of multiple births had a limited impact on national changes in stillbirth rates.

To exclude any potential effects of the COVID-19 pandemic on stillbirth rates, we performed the decomposition between 2010 and 2015 with similar results (Supplementary Fig. S3).

### Decomposition of stillbirth rates between countries

With the exception of Belgium, where the national stillbirth rate is comparatively high due to the inclusion of TOPs, the differences in rates range from being 0.71 higher than average in Latvia to 1.30 per 1000 lower than average in Finland (Fig. 4). Having a maternal age structure that is at higher risk than average (e.g. higher proportion of advanced-age mothers) is contributing to a higher than average national rate of up to 0.13 per 1000 in Ireland. Having a maternal age structure that is at lower risk than average is contributing to a lower than average national stillbirth rate of up to 0.11 per 1000 in Belgium.

The comparatively high prevalence of multiple births in Cyprus contributes substantially to its higher than average stillbirth rate in 2021. This makes Cyprus an outlier as multiplicity otherwise only has a minor impact on a country’s deviation from the European average.

Overall, differences in maternal age and multiplicity structure explain only a small part of the variation in stillbirth rates between countries. To exclude the effect of the COVID-19 pandemic, we performed a decomposition between countries for 2015 (Supplementary Fig. S4). Effects of differences in maternal age and multiplicity were similar.

## Discussion

We examined trends and differences in stillbirth rates across European countries to understand the impact of variation in maternal age structure and multiplicity. While these factors explain some national trends, they leave most variation in stillbirth rates over time and between countries unexplained.

As the risk of stillbirth across maternal age is J-shaped, with a higher risk for teenage pregnancies and older mothers [10, 11], the change in maternal age structure can have two effects. An increase in advanced-age mothers can lead to a higher proportion of high-risk pregnancies. A decrease in teenage pregnancies can also lead to a decrease in the number of high-risk pregnancies. This interpretation only holds under fairly strong assumptions, as the relationship between age and stillbirth is highly confounded [28]. For the decomposition of national trends, we observe positive effects of the changing maternal age structure for 16 out of 24 countries, i.e. the increase in advanced age alone would have increased the national stillbirth rate. This effect is the greatest in countries with a large increase in pregnancies among older mothers. For 6 out of 24 countries, the change in age structure had rate-reducing effects due to the reduction in teenage pregnancies. This leads to a reduction in high-risk pregnancies, which results in a decrease in the national stillbirth rate. Multiple births decreased over time in 18 out of 24 countries. For these countries, the decomposition of the trends over time showed that if only the reduction in multiple births would have occurred, this would have reduced the national stillbirth rate. However, as the proportion of multiple births is quite low in all countries, the effect size is small. For the average country, the increase in pregnancies at advanced maternal age contributed to an increase in national stillbirth rates between 2010 and 2021, regardless of how group-specific rates might change. At the same time, the reduction in multiple births contributed to a decrease in national stillbirth rates.

Maternal age and multiplicity only explained a small part of the differences between countries in 2021, with some exceptions. However, a higher than average multiple birth rate may explain a higher than average stillbirth rate, as observed for Cyprus, but overall the effect sizes are small as the multiple birth rate becomes more uniform in 2021 (except Cyprus). A higher than average proportion of teenage and advanced maternal age pregnancies explained small proportions of the differences to the cross-country average in 8 out of 24 countries. A lower than average risk age structure also explained little of the variation, accounting for 14 out of 24 countries. The lack of explanation for maternal age structure is due to an increasingly homogeneous age structure between countries, as well as the decreasing importance of maternal age as a risk factor over time. We observed a sharp decrease in the risk of stillbirth for older mothers between 2010 and 2021 (Supplementary Fig. S2). As maternal age is less important as a risk factor in 2021, differences in the age structure between countries affect national differences in stillbirth rates to a smaller extent. As pregnancies at advanced ages have become more common, more research has been devoted to understanding and managing these cases. As a result, health professionals have gained more experience in dealing with those pregnancies. Increased knowledge of complications can also improve monitoring and the use of interventions when necessary. For example, new research advises offering inductions of labour to advanced-age mothers to prevent late stillbirth, particularly in term and late-term pregnancies [29]. As a result, the number of inductions of labour for women aged 40 has increased during the last 20 years in Iceland [30].

Although maternal age and multiplicity could explain some of the national trends and differences between countries, the larger proportion remains unexplained by these structural factors. Decreasing stillbirth rates could be attributed to improvements in antenatal and obstetric care and better understanding of behavioural aspects, such as self-monitoring of foetal movements [31] or a reduction in maternal smoking rates [32]. Differences in the level of stillbirth rates could be explained by differences in the quality of, and access to, antenatal and obstetric care. These differences may also be influenced by different guidelines for induction of labour in high-risk pregnancies [33]. Decreases in stillbirth rates may also result from increased TOP of foetuses with life-threatening conditions, who are more often identified due to improved antenatal screening. Differences in access to TOP may have also influenced our results. For example, in Malta, where TOP is illegal, pregnancies with life-threatening foetal conditions must be continued, possibly leading to higher stillbirth rates compared to countries where TOP is permitted. Since pregnancies resulting from assisted reproduction have a higher risk of stillbirth than spontaneous conceptions [34], differences in the use of assisted reproduction can impact the differences in trends and levels of stillbirth rates. However, Euro-Peristat does not collect data on assisted reproductive treatment, and national birth registries often lack reliable information on it.

Hidden structural changes can also explain the remaining differences, as there are more risk factors for stillbirth than just maternal age and multiplicity. Changes in maternal conditions such as hypertension, diabetes, or obesity [35], but also changes in the proportion of births to migrant mothers could explain some of the differences in stillbirth rates [36].

Our study provides valuable insights into how maternal age and multiple pregnancies affect stillbirth rates across 25 countries. The data quality is high as we use routine sources which are validated for several countries [37, 38]. The method we use allows us to analyse our research question when only aggregated data is available. We also account for differences in group-specific mortality rates. Countries with more pregnancies at advanced maternal age may have better infrastructure and guidelines to reduce stillbirth risk than those where such pregnancies are rare. The decomposition method uses group-specific average stillbirth rate to account for these differences.

However, our study has some limitations. The results for Belgium, Ireland, and Cyprus should be interpreted with caution, as they cannot exclude TOP from stillbirth data, making comparisons difficult. Data since 2020 is influenced by the COVID-19 pandemic. However, sensitivity checks show that this is not changing the impact of the compositional factors analysed. We analysed maternal age and multiplicity separately, but these factors interact. Multiplicity is more likely with increasing maternal age and assisted reproduction, which is also more common among older women [19]. Nonetheless, our results remain valid when analysed separately.

## Conclusion

The increase in pregnancies at advanced maternal age, which is associated with a higher risk of stillbirth, contributes to an increase in national stillbirth rates over time. In addition, the reduction in multiple births contributes to a decrease in national stillbirth rates over time. However, large parts of those trends remain unexplained. By 2021, maternal age and multiple births no longer explain major differences between countries, due to the increasing homogeneity of these factors and the decreasing risk of stillbirth in advanced maternal age.

## Supplementary Material

ckae214_Supplementary_Data

## Data Availability

Due to national data protection guidelines, data is confidential. R-code is available on GitHub: https://github.com/mskniffka/peristat_24. Key points The increase in pregnancies at advanced maternal ages, which is connected to a higher risk of stillbirths, contributes to an increase or dampens the decrease in national stillbirth rates between 2010 and 2021. The reduction in the prevalence of multiple births in 25 European countries since 2010 contributed to improvements in stillbirth rates in most countries. The trends in stillbirth rates and rate differences between European countries are only partially explained by changes in maternal age at pregnancy or multiplicity, with most of the rate differences remaining unexplained. In 2021, maternal age and multiplicity had low explanatory power for between-country differences due to countries becoming more similar along these strata and the decreasing risk of stillbirth at advanced maternal age.
