# Supplementary material for: Stillbirth rate trends across 25 European countries between 2010 and 2021: the contribution of maternal age and multiplicity
Source: Eur J Public Health. 2025 Jan 21;35(2):319–27. doi: 10.1093/eurpub/ckae214 (PMC11967907; doi:10.1093/eurpub/ckae214)
Supplement: ckae214_Supplementary_Data [file ckae214_supplementary_data.pdf]

Supplementary materials

Country	Annual change in stillbirths per 1,000 births (95% CI)	Breakpoint year	Breakpoint test p-value	MK-trend test p-value
Austria	0.003 (-0.027; 0.034)	-	0.124	0.845
Belgium	0.062 (0.017; 0.106)	-	0.541	0.020
Croatia	-0.040 (-0.111; 0.032)	-	0.602	0.307
Cyprus	0.006 (-0.136; 0.147)	-	0.888	0.945
Czech Rep.	0.037 (0.005; 0.070)	-	0.107	0.776
Denmark	-0.045 (-0.091; 0.001)	-	0.538	0.084
Estonia	-0.133 (-0.194; -0.072)	-	0.195	0.001
Finland	-0.012 (-0.037; 0.013)	-	0.064	0.375
France	0.006 (-0.018; 0.030)	-	0.728	0.749
Germany	0.056 (0.042; 0.069)<br>0.143 (0.104; 0.181)	2018	0.008	0.002
Iceland	0.059 (-0.065; 0.183)	-	0.238	0.267
Ireland	-0.066 (-0.117; -0.015)	-	0.171	0.561
Italy	-0.011 (-0.026; 0.004)	-	0.813	0.184
Latvia	-0.128 (-0.197; -0.059)	-	0.264	0.111
Lithuania	-0.108 (-0.165; -0.052)	-	0.574	0.004
Luxembourg	0.075 (-0.078; 0.228)<br>-0.562 (-0.804; -0.320)	2018	0.049	0.053
Malta	-0.074 (-0.257; 0.109)	-	0.554	0.451
Netherlands	-0.186 (-0.227; -0.145)<br>0.133 (0.064; 0.201)	2017	0.004	0.704
Norway	-0.084 (-0.122; -0.046)	-	0.506	0.002
Poland	-0.105 (-0.148; -0.063)<br>0.225 (0.033; 0.416)	2019	0.044	0.002
Slovenia	-0.063 (-0.157; 0.030)	-	0.643	0.222
Spain	-0.055 (-0.073; -0.036)	-	0.079	0.029
Sweden	0.045 (-0.080; 0.169)<br>-0.100 (-0.143; -0.057)	2014	0.028	0.004
Switzerland	-0.011 (-0.037; 0.014)	-	0.631	0.409
UK	-0.109*** (-0.142; -0.075)	-	0.159	0.280

Table S1: Estimated annual changes in stillbirths per 1,000 births across 25 European countries between 2010 and 2021 (2013 to 2021 for the UK and Slovenia, 2014 to 2021 for France and 2015 to 2021 for Luxembourg). A single rate of change is reported unless a significant breakpoint in the linear trend was identified. For countries with significant breakpoints we report the rate of change in stillbirths prior and after the break. The year of the breakpoint is annotated. P-values are given for the breakpoint test and the Mann-Kendall test for the presence of a monotonic trend. Source: Euro-Peristat Network.

Country	Source	TOP excluded
Austria	Statistics Austria	yes
Belgium	Statistics Belgium (Statbel)	no
Croatia	Croatian Public Health Institute	yes
Cyprus	The Health Monitoring Unit, Cyprus Ministry of Health	no
Czechia	Czech statistical office, Information system of births	yes
Denmark	Statistics Denmark	yes
Estonia	Estonian Medical Birth Register, Linked Data from EMSR (Medical Birth) and SPR (Causes of Death), National Institute for Public Health	yes
Finland	Medical Birth Register (THL Finnish Institute for Health and Welfare	yes
France	qualiN by Inserm	yes
Germany	IQTIG	yes
Iceland	The Icelandic Birth Registration (IBR)	yes
Ireland	NPRS, Healthcare Pricing Office	no
Italy	National Institute of Statistics of Italy (ISTAT)	yes
Latvia	The Medical Birth Register and Register of Cause of Death, The Centre for Disease Prevention and Control of Latvia	yes*
Lithuania	Institute of Hygiene Health Information Centre (HI HIC)	yes
Luxembourg	Luxembourg Institute of Health	yes
Malta	MINISTRY FOR HEALTH AND ACTIVE AGEING	yes
Netherlands**	Perined	yes
Norway	Norwegian Institute of Public Health	yes
Poland	Central Statistical Office	yes
Slovenia	Vital statistics, Central Statistical Office	yes
Spain	Vital statistics, Central Statistical Office	yes
Sweden	The National Board of Health and Welfare	yes
Switzerland	FSO, Federal Statistical Office, Switzerland: Swiss Vital Statistics (BEVNAT) and Cause of death statistics (CoD) © BFS	yes
UK	MBRRACE-UK	yes

*TOP are excluded in Latvia from 2017, but should be very rare before 2017 since they are only allowed until 24 weeks of gestation in special cases.
**Due to registration problems, foetal deaths in 2021 might be under-recorded in the Netherlands.

Table S2: Data sources.

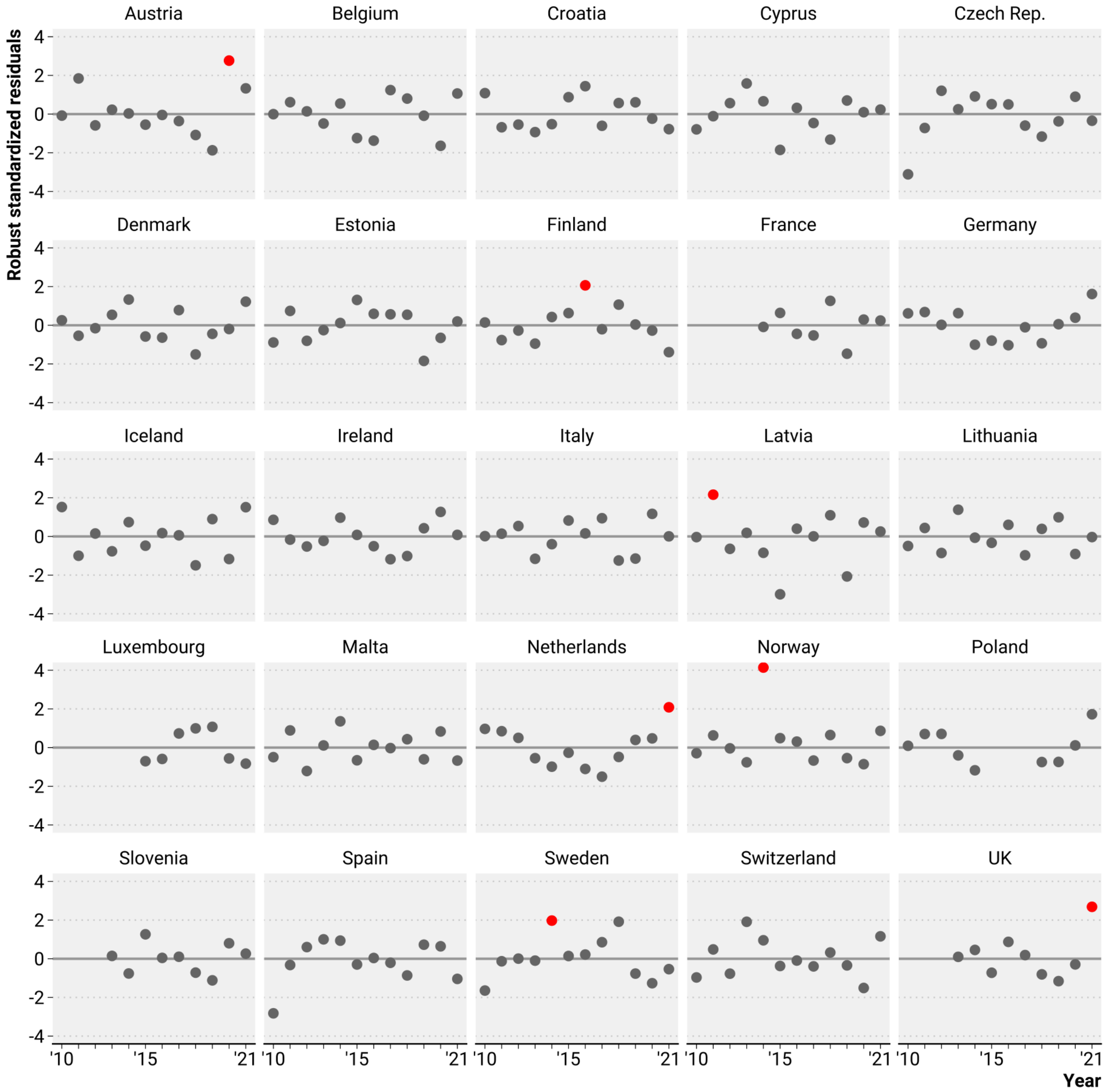

Figure S1: Annual standardized residuals from robust linear fit of stillbirths on year. Outliers with z>2 marked in red.

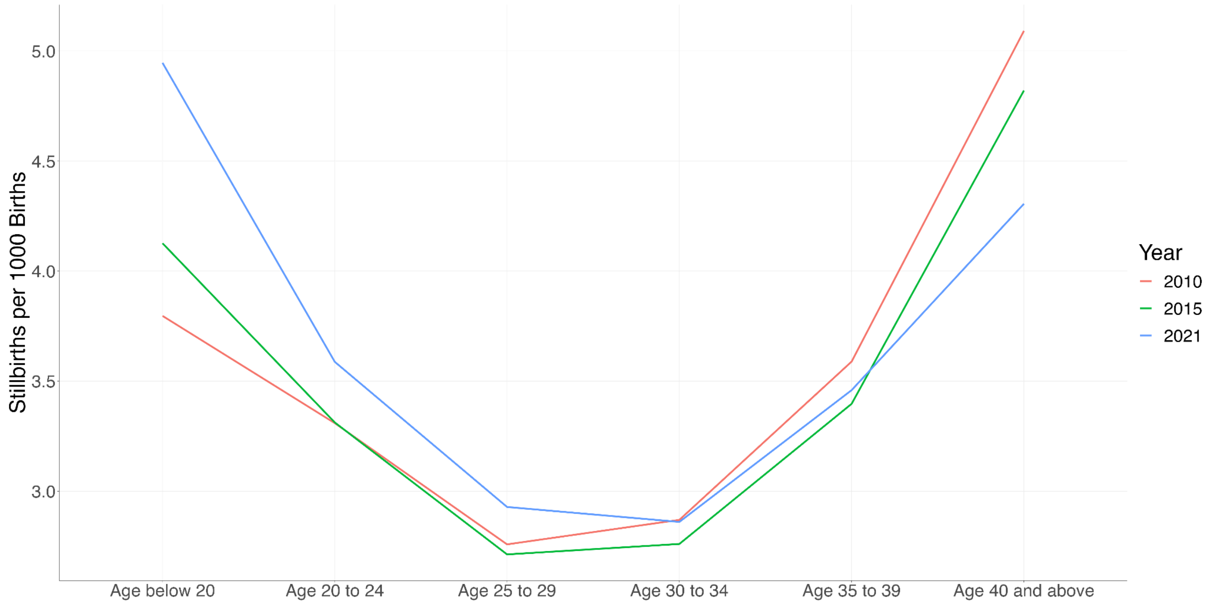

Figure S2: Shape of stillbirth rates by age over time from 2010 to 2021. Data is aggregated from all countries except Cyprus, Ireland and Belgium due to inclusion of TOP and UK, Slovenia and France due to missing data in 2010. Source: Euro-Peristat Network.

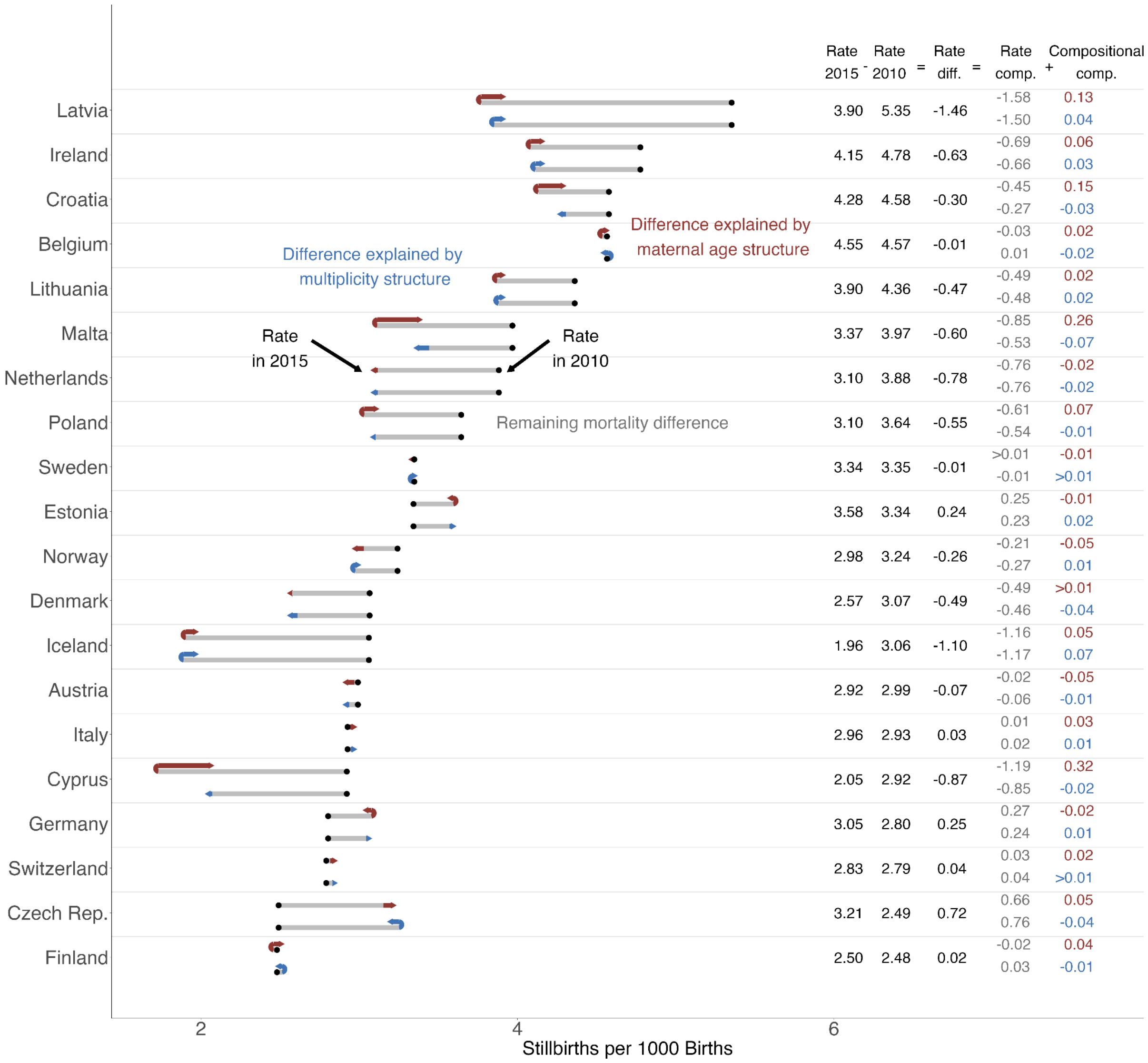

Figure S3: Decomposition of the difference in stillbirth rates by country between 2010 and 2015 into the component of changing stillbirth rates (grey), changing maternal age composition (red) and changing prevalence of multiples (blue). Decomposition of maternal age structure and multiples were performed separately. The black dot represents the rate in 2010 and the end of the arrow represents the rate in 2021. Due to missing data in 2010, France, Slovenia, the UK and Spain had to be excluded from the decomposition. Source: Euro-Peristat Network.

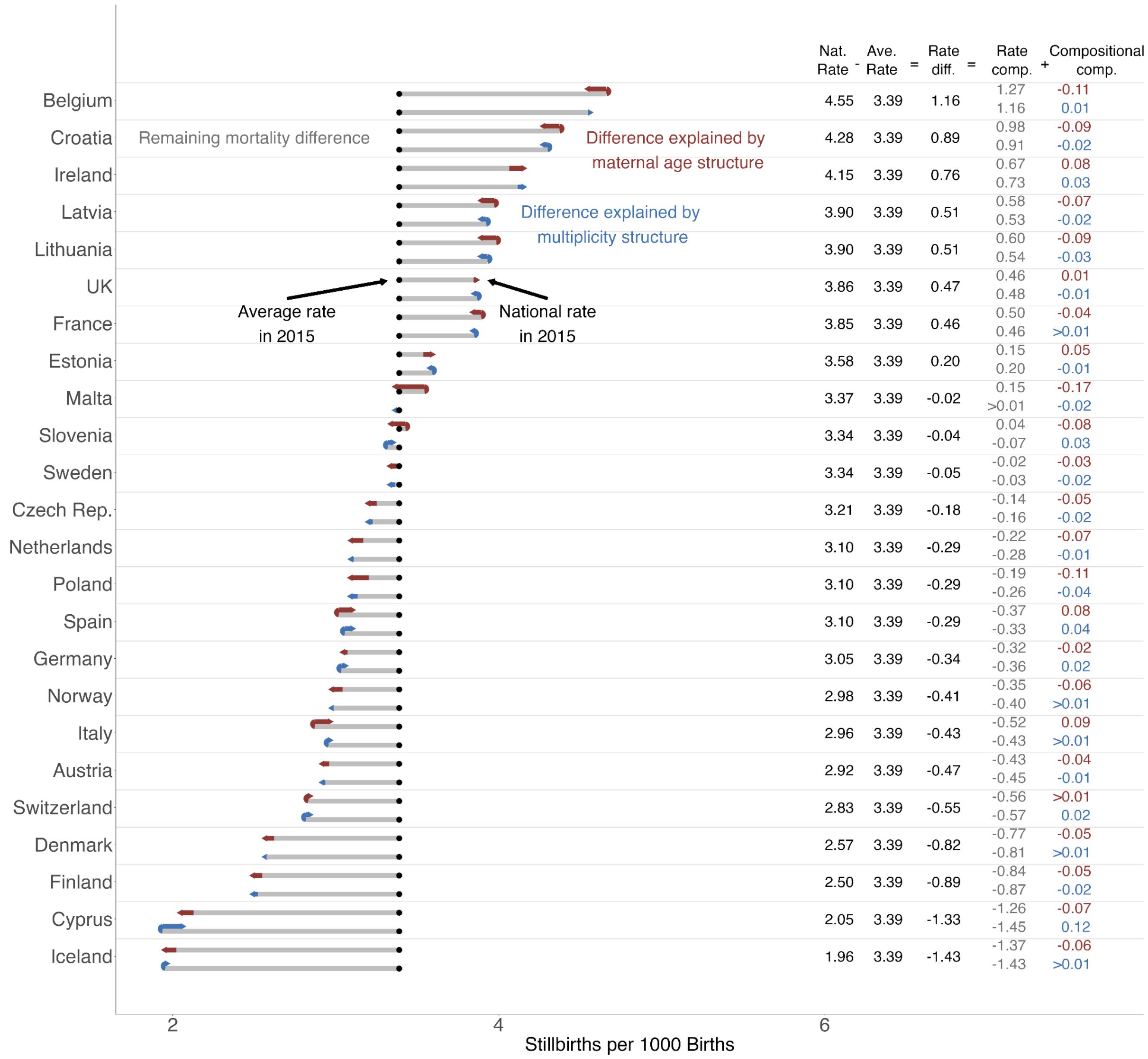

Figure S4: Decomposition of the difference in stillbirth rates between countries and European average in 2015 into the component of changing stillbirth rates (grey), changing maternal age composition (red) and changing prevalence of multiples (blue). Decomposition of maternal age structure and multiples were performed separately. The black dot represents the average rate in 2015 across all countries and the end of the arrow represents the national rate in 2015. Source: Euro-Peristat Network.
